# Feasibility and acceptability of phone-delivered psychological therapy for refugee children and adolescents in a humanitarian setting

**DOI:** 10.1186/s13031-023-00565-2

**Published:** 2024-01-13

**Authors:** Fiona S. McEwen, Hania El Khatib, Kristin Hadfield, Karen Pluess, Nicolas Chehade, Tania Bosqui, Stephanie Skavenski, Laura Murray, Roland Weierstall-Pust, Elie Karam, Michael Pluess

**Affiliations:** 1 Biological and Experimental Psychology, School of Biological and Behavioural Science, G.E. Fogg Building, Queen Mary University of London, Mile End Road, London, UK; 2 Department of War Studies, King’s College London, Strand, London, UK; 3 Trinity Centre for Global Health, School of Psychology, Trinity College Dublin, Dublin, Ireland; 4 Médecins du Monde, Beirut, Lebanon; 5 Department of Psychology, American University of Beirut, Beirut, Lebanon; 6 Johns Hopkins Bloomberg School of Public Health, Baltimore, MD USA; 7 Medical School Hamburg, Hamburg, Germany; 8 Institute for Development, Research, Advocacy and Applied Care, Achrafieh, St. George Hospital Street, Beirut, Lebanon; 9 Saint Georges Hospital University Medical Center, Achrafieh, Beirut, Lebanon; 10 Saint Georges University of Beirut, Beirut, Lebanon; 11 School of Psychology, University of Surrey, Guildford, UK

**Keywords:** Telepsychiatry, Telephone, Children and adolescents, Refugees, Feasibility and acceptability, Lay counsellors

## Abstract

**Background:**

Refugee children are at high risk of mental health problems but face barriers to accessing mental health services, a problem exacerbated by a shortage of mental health professionals. Having trained lay counsellors deliver therapy via telephone could overcome these barriers. This is the first study to explore feasibility and acceptability of telephone-delivered therapy with refugee children in a humanitarian setting.

**Methods:**

An evidence-based intervention, Common Elements Treatment Approach, was adapted for telephone-delivery (t-CETA) and delivered by lay counsellors to Syrian refugee children in informal tented settlements in the Beqaa region of Lebanon. Following delivery of t-CETA, semi-structured interviews were conducted with counsellors (N = 3) and with children who received t-CETA (N = 11, 45% female, age 8–17 years) and their caregivers (N = 11, 100% female, age 29–56 years) (N = 25 interviews). Thematic content analysis was conducted separately for interviews with counsellors and interviews with families and results were synthesized.

**Results:**

Three themes emerged from interviews with counsellors and four themes from interviews with families, with substantial overlap between them. Synthesized themes were: counselling over the phone both solves and creates practical and logistical challenges; t-CETA is adapted to potential cultural blocks; the relationship between the counsellor and the child and caregiver is extremely important; the family’s attitude to mental health influences their understanding of and engagement with counselling; and t-CETA works and is needed. Counselling over the phone overcame logistical barriers, such as poor transportation, and cultural barriers, such as stigma associated with attending mental health services. It provided a more flexible and accessible service and resulted in reductions in symptoms for many children. Challenges included access to phones and poor network coverage, finding an appropriate space, and communication challenges over the phone.

**Conclusions:**

Despite some challenges, telephone-delivered therapy for children shows promising evidence of feasibility and acceptability in a humanitarian context and has the potential to increase access to mental health services by hard-to-reach populations. Approaches to addressing challenges of telephone-delivered therapy are discussed.

*Trial Registration* ClinicalTrials.gov ID: NCT03887312; registered 22nd March 2019.

**Supplementary Information:**

The online version contains supplementary material available at 10.1186/s13031-023-00565-2.

## Background

An unprecedented number of people are displaced due to conflict or persecution, with United Nations High Commissioner for Refugees (UNHCR) estimating that over 100 million people are forcibly displaced, 41% of whom are children [1, 2]. The majority of displaced people live in low- and middle-income countries, often in camps and informal settlements [2, 3]. Many have been exposed to war but also face ongoing adversities, including limited access to basic resources, housing, education, and health-related services. These cumulative stressors contribute to a significantly elevated risk of associated and often chronic mental health problems and also prevent access to vital treatment [3].

The Syrian civil war led to millions being displaced internally and into surrounding countries. By early 2022, more than five million Syrians were registered as refugees in neighboring countries [4]. Nearly a million are registered in Lebanon: a majority live in the Beqaa region with nearly half in tents or other non-permanent shelters [5, 6]. Few anticipate a return to Syria and most are likely to live in these conditions for the foreseeable future [7]. Approximately half of children living in informal settlements in Beqaa are estimated to meet criteria for at least one mental disorder [8]; however, mental health service provision is limited and access is challenging. Amongst refugee families in Beqaa, less than 10% who express interest in mental health services for their child end up accessing treatment (Pluess et al., in preparation) and similar problems have been reported for adults in this region [9]. Commonly reported barriers include long distances to clinics, lack of transportation, expense, concerns about safety when travelling (risk of violence or being stopped at military checkpoints when many Syrian refugees lack necessary legal documentation), and lack of knowledge about which services to approach and where these services are located [9, 10]. Furthermore, access to services has been restricted due to the COVID-19 pandemic response; extreme weather events, such as flooding and heavy snow; and civil unrest resulting in widespread road closures. Thus, there is a fundamental need for accessible, effective, and efficient evidence-based mental health treatments for refugees, as well as vulnerable host communities who face similar barriers.

Delivering services via telephone provides one potential solution to increase access when mobility is restricted and resources are sparse. A recent meta-analysis found that remote delivery of therapies, such as via video calls or telephone, can be effective for young people [11]. Access to the technology required for video calls (e.g., smartphones, software, and bandwidth) is often limited in humanitarian settings, so the use of standard telephone calls may have wider applicability. To establish the potential for telephone-delivered therapy to overcome access barriers in humanitarian settings, it is essential to understand whether it is feasible and acceptable both to those providing and those receiving services over the phone. Although there has been a telemedicine expansion during the COVID-19 pandemic [12], there is a dearth of literature on telepsychiatry in low- and middle-income countries or settings such as refugee camps [13] and it is not yet clear whether telephone-delivered therapy is feasible or acceptable in these contexts.

A further challenge in humanitarian and resource-constrained settings is a lack of mental health professionals to deliver interventions [14]. However, trained and supervised lay counsellors have been found to be able to effectively deliver face-to-face evidence-based psychological therapies, including transdiagnostic interventions such as Common Elements Treatment Approach (CETA) and Early Adolescents Skills for Emotions (EASE) [15–17]. CETA has been developed specifically for use with lay counsellors in low- and middle-income countries, with evidence of effectiveness in adults [18–20] and preliminary evidence for effectiveness in children [21].

## Method

### Aim

In this study we focus on the use of CETA specifically adapted for delivery via telephone – t-CETA – and delivered by trained and supervised lay counsellors, with the aim of exploring whether this is a feasible and acceptable approach to delivering mental health services to children in a humanitarian setting. We do this through thematic content analysis of interviews with counsellors who delivered t-CETA, as well as interviews with children who received t-CETA and their caregivers, asking: (1) *What are the perspectives of counsellors on the delivery of t-CETA to Syrian refugee children over the telephone?* (2) *How did Syrian refugee children and their caregivers experience receiving psychological treatment (t-CETA) over the telephone?* This qualitative approach was chosen to provide an in-depth understanding of potential success factors but also challenges related to the implementation and application of telephone-delivered therapy. To the best of the authors’ knowledge, this is the first study that has systematically studied the feasibility and acceptability of telephone-delivered therapy in a sample of health workers and clients from a humanitarian setting. Data on effectiveness and mechanisms of change of t-CETA are described separately [22, 23].

### Setting

This paper describes the qualitative component of a mixed-methods randomized controlled trial (RCT) of t-CETA (ClinicalTrials.gov ID: NCT03887312 [24]) carried out May-December 2019. Ethical approval was granted by the Institutional Review Board of the American University of Beirut (ref: SBS-2017–0429) and the study was approved by the Ministry of Public Health in Lebanon (ref: 2017/4/49165). Participants in the RCT were Syrian families living in informal tented settlements (ITS) in the Beqaa region of Lebanon.

### Intervention

The therapy delivered via phone was Common Elements Treatment Approach (CETA; [15, 21, 25]). CETA is an established transdiagnostic intervention for children with common mental health problems and incorporates evidence-based treatments (mostly using cognitive behavioral therapy [CBT] approaches) into one package, including treatments for depression, anxiety, trauma-related symptoms, externalizing behavior problems, and substance use. CETA can be delivered by lay counsellors after comprehensive training [26]. Most components are delivered to the child, with content repeated with the caregiver to enable them to support their child to complete homework; however, the component for externalizing behavior problems is delivered only to caregivers. For the current study, the original CETA manual underwent linguistic and cultural adaptation for Syrian children, while retaining the essence and content of CETA.

CETA was adapted to be delivered via telephone (t-CETA) jointly by the local team at Médecins du Monde in Lebanon and the CETA team at Johns Hopkins University. Adaptations included shorter, more frequent sessions; strategies for maintaining child engagement over the phone, such as gamifying the content; alternatives to workbooks and written materials; and training counsellors to focus on non-visual cues [27]. Safety protocols were developed in consultation with child protection and gender-based violence experts who work with the target community.

### Recruitment and training of lay counsellors

Lay counsellors were recruited to deliver the intervention: requirements included education to bachelor degree level and experience in social work/case management in humanitarian settings with Syrian refugees. The recruitment process included role-plays to evaluate aptitude for counselling, including the ability to deal appropriately and sensitively with issues such as disclosure of maltreatment or self-harm/suicidal ideation. Lay counsellors completed a six-day CETA training course delivered by a CETA expert (SS), followed by an eight-week period of training and practice sessions led by a local psychotherapist (NC). Sessions included roleplay of CETA components and feedback from the supervisor, and education about common mental disorders and how they present in children (two-to-three sessions per week, three-to-four hours per session). Following this, the counsellors delivered CETA under the supervision of the local psychotherapist, who was in turn under the supervision of the CETA expert.

### Participants

Semi-structured interviews were conducted with (1) the two lay counsellors who delivered t-CETA and the supervisor who directly oversaw delivery (all three referred to as counsellors throughout, for simplicity), and (2) 11 children who received t-CETA and their primary caregivers. The counsellors were Lebanese, two females and one male, aged 25–29 years, and had a bachelor’s degree (counsellors) or were a clinical psychologist in training (supervisor). Children and adolescents were eligible for the RCT if aged 8–17 years and had a diagnosis of a common mental disorder (depression, anxiety disorder, PTSD, conduct or oppositional defiant disorder). Full inclusion criteria for the RCT are described in Additional file 1. Children and caregivers were eligible to take part in the semi-structured interviews if the child had fully or partially (≥ 2 sessions) completed a course of t-CETA. Of N = 12 children who met these criteria, N = 11 participated (n = 8, full course; n = 3, partial course). In all cases, both the caregiver and child took part. The number of sessions via phone ranged from 2–12 (*M*[*SD*] = 7.8 [4.1]). Five children (45%) were female and six (55%) were male, aged 8–17 years (*M*[*SD*] = 10.9 [2.6]). All 11 caregivers were female (10 mothers and one grandmother), aged 29–56 years (*M*[*SD*] = 36.4 [8.4]). All families lived in informal tented settlements.

### Procedure

Counsellors were invited to take part in semi-structured interviews after completion of the RCT. After providing informed consent, semi-structured interviews were conducted online in English by KH between December 2019 and February 2020. Interviews lasted 54–59 min (*SD* = 2.65). Interview questions related to delivery of CETA to refugee children in person and via phone, benefits and challenges of working with a Syrian refugee population, program efficacy, how counsellors worked through challenges they faced, and training and supervision. Interviews were audio recorded, transcribed, and identifying details were removed (see Additional file 1). No remuneration was provided to counsellors for participation.

Families that received t-CETA were approached by phone after completion of the RCT. Information about the study was read to families over the phone and verbal consent taken from caregivers; verbal assent was taken from children only if their caregiver provided consent. Financial compensation for participants’ time was provided. Semi-structured interviews were conducted over the phone in Arabic by HEK, during November and December 2019. Caregiver interviews lasted 20–57 min**,** and child interviews lasted 20–33 min (total time per family, *M*[*SD*] = 56.64 [13.76]). Children and caregivers were interviewed individually, with the exception of one 11-year-old who was more comfortable being interviewed together with their caregiver. Interview questions related to the experience of receiving telephone-delivered therapy, the extent of improvement of the child’s problems, session content, relationship with the counsellor, and benefits and challenges of receiving therapy over the phone (see Additional file 1). Interviews were audio-recorded, transcribed, and translated to English, and identifying information removed. Names have been replaced with pseudonyms throughout.

### Analysis

Thematic content analysis was conducted independently for the counsellor interviews and the child and caregiver interviews (Braun & Clarke, 2006). Interviews with counsellors were transcribed, reviewed, and coded by KH. Interviews with children and caregivers were transcribed and translated by HEK, reviewed for accuracy by Syrian and Lebanese researchers, then reviewed and coded by HEK. Data were classified into smaller meaningful categories through a process of open coding [28].

Data relevant to the research questions were coded inductively line-by-line by one author (KH, counsellor interviews; HEK, child and caregiver interviews), with codes inductively deriving from the content of the data as opposed to the researchers’ presuppositions. For counsellor interviews, all codes were reviewed by KP, and for child and caregiver interviews, codes were reviewed by KH and/or FM for 55% of interviews. Reviewing authors commented on the classification of text and category labels assigned to them; codes were revised where necessary. Codes were reexamined, related codes grouped together, and these codes sorted into potential themes [29]. To promote credibility in the analysis, after theme development, the authors who assisted with coding also assisted with the refinement of themes. Researcher triangulation was employed, with regular meetings to discuss coding and emerging themes, as well as refinement of the final themes together. This was an iterative process, and disagreements in coding and themes were discussed to achieve consensus [30].

## Results

**Fig. 1:**
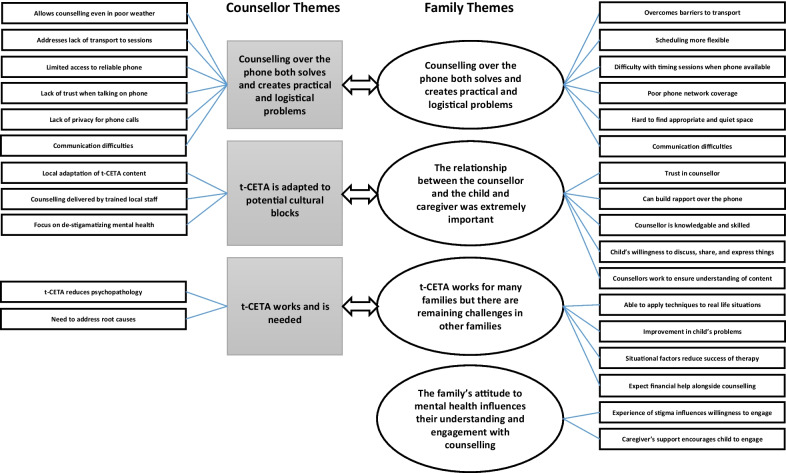
Results of thematic content analysis of interviews with counsellors and families (individual interviews with children and caregivers)

Three themes emerged from analysis of counsellor interviews. The counsellors believed that (1) counselling over the phone both solves and creates practical and logistical problems, (2) t-CETA is adapted to potential cultural blocks, and (3) t-CETA works and is needed. Four themes emerged from analysis of child and caregiver interviews: (1) counselling over the phone both solves and creates practical and logistical problems, (2) t-CETA works for many families but there are remaining challenges in other families, (3) the relationship between the counsellor and the child and the caregiver is extremely important, and (4) the family’s attitude to mental health influences their understanding of and engagement with counselling. While the analyses were conducted separately, there was significant overlap between themes (Fig. 1) and so results are presented by theme, highlighting similarities and differences between the perspectives of the counsellors and families where relevant.

### Counselling over the phone both solves and creates practical and logistical challenges

#### Solving logistical issues

The counsellors identified a number of challenges that refugees face in accessing traditional, face-to-face counselling, largely centered on difficulties travelling to a clinic. Because of the pervasive poverty of many Syrian refugees in Lebanon, few own a car and the cost of using taxis or buses is prohibitive. Furthermore, travelling to and from the clinic takes time and may not be possible for those with caring responsibilities. One of the counsellors explained,

Many of the families in the area that we work are in need therapy but cannot come to the clinic because of lack of transportation or because maybe the mother had many kids and they had to stay with them in the tent or maybe someone is sick and cannot move from the tent or maybe it’s far away from them.

Many families echoed these issues. Caregivers described the difficulty of getting to a clinic and the comparative ease of receiving treatment over the phone, describing the expense of transport, time taken to travel, and difficulties when there are other children to take care of or when children attend school. A mother, comparing travelling to the clinic before starting phone sessions, said, “it takes time going and coming. But over the phone, we do the session and that’s it, it does not take a lot of our time.” This point was also made by several children. One mother said that it is not safe to leave other children at home in a camp, and another explained, “I preferred them over the phone, because you know I have young kids … If I went I would have to leave them … on the phone they can be beside me.”

In addition, counsellors explained that parents found it hard to leave the settlement because of fear of “if something happened on the road”, “if a fire happened” in the settlement, or “the danger of being held at a checkpoint” without papers. The risk of sexual violence also impacted the mobility of women and children: “they heard that a woman got harassed by a taxi driver. Everyone stopped going in taxis”. Finally, severe weather conditions were a barrier to accessing in person counselling. A counsellor explained, “Their clothes are not equipped for cold – intense cold – in Beqaa for example. And the roads are not equipped for rain, and for flooding… for snow.” Counsellors pointed out that when a family misses a traditional in-person appointment, typically they would have to wait a week or two until their next scheduled session. If the barriers to attending are unchanged at that point, they will miss the rescheduled appointment too. This leads to many missed in-person appointments. Telephone delivery was seen by counsellors as a way to reach more and harder-to-reach children, to be more logistically flexible, to hold more appointments, and to be able to follow up more easily and rapidly when sessions were missed. This flexibility was also highlighted by mothers who described the counsellor calling to remind them of the appointment the day before and then again in the morning; if it was a bad time when the counsellor called – for example, because the family was out – then the counsellor would call back later to do the session.

#### Logistical challenges

Although t-CETA addressed the above problems, counsellors identified that phone-delivery created its own challenges which then had to be addressed. First, many children did not have reliable access to a phone. While some caregivers indicated that this was not a problem because they had two phones or borrowed one from a relative, in many families there was only one phone available due to the “very, very high telephone costs” (counsellor). This phone was typically with the father, potentially leading to issues when the father was at work; “whenever I call, I have to wait for the father until he goes back to the home” (counsellor). To address this, where possible the counsellors would get phone numbers for relatives or neighbours, and try to hold the sessions over this alternative phone. This worked where families felt comfortable with their relative or neighbour knowing that the child was in counselling, but some families wanted to keep this private. A counsellor explained one case where,

The mother refused at all to use her neighbour’s phone because sometimes think they will record it … She didn’t feel safe to use the neighbour’s phone, so we stopped. We cannot do anything so we stopped the counselling with the child, but as a good thing that happened, after one month and a half the father came back to their home and he started working beside the home so we continued again.

Even where access to a phone was consistent, network coverage and phone charging were not always reliable. A counsellor said, “Too many times when I was calling the family, the phone will be off or they lose the battery or we had the bad connection.” Some families also highlighted this. One mother said, “The phone would disconnect a lot. Because … the coverage is very weak … Like now I have to keep my phone always outside, I don’t want to stay sitting outside in the cold so I get coverage.” Counsellors said that this could be a major problem, because when a call was dropped then “we have to call again and call again” and each time the child and counsellor would have to repeat what they were saying, some of which was “very, very sensitive” information which the children would not want to repeat. This slowed some sessions and interrupted their flow.

A second issue that phone delivery raised was in the safety of talking on the phone. A counsellor explained that “the Syrian refugees are very wary of using the phone because their government … spies on people over the phone.” Some families were worried about the government spying on them, or their relatives or neighbours secretly recording their phone calls.

A third issue was lack of privacy. Many refugees live in one or two room tents, often with large families and in close proximity to other tents. One counsellor said, “In a tent, you can hear everything around you. So there’s noise always. Sometimes there’s no doors in the tent, so someone will enter and go during the session.” The tents where the children live are “the least private space ever … if you’re whispering in the room, you’ll hear it in another room” (counsellor). The counsellors would explain to the parents and to the child that the counselling should be done in private, and in most cases, the families tried to respect this despite the logistical difficulties. However, it was not always possible. For example, one mother said it was, “hard for [the counsellor] to hear us and us to hear her … my daughter would go to the other room but you know it’s a tent... as much as you move away you will hear sound.” The counsellors explained that the therapy was less likely to be effective when there is lack of privacy, since the children may not feel comfortable sharing their experiences and feelings in the presence of others.

However, not all families reported these problems and some discussed finding a private space to do the sessions: “I empty the space for him and I give him [the phone] to speak,” (mother); “I sit alone, and we talk, her [counsellor] and I, just me alone … And when she wants my mom, I go out and give her and my mom sits alone” (child).

#### Communication over the telephone

Councillors reported some difficulties in comprehension and communication over the phone. In-person, the counsellor “could use a board to explain or the sheets or a material in my hand to explain for the child”, but over the phone they had to explain concepts to the children with no aids. This was challenging because the counsellors would give “examples for shapes or numbers or timeline” in the sessions but these concepts were new to many children because they had had limited access to school. The counsellors had to teach the children these concepts before they could begin the therapy work itself: “after many efforts we could provide the therapy that we were providing. But it took many efforts.” However, the counsellors indicated that it was possible to teach these concepts to the children over the phone and that after this, therapy could begin.

In-person therapy involves both visual and auditory information, but telephone therapy is solely auditory and so some information is lost. Aside from the lack of aids, the impacts of a lack of visual information were viewed to be mixed by the counsellors. The counsellors indicated that this could be a major positive; one felt that children trusted them more because the children could never see any judgement in their expression, and that not being in person allowed the children to “feel free to talk about everything.” It also could be a challenge though, with another counsellor indicating that they could not tell “from his facial expression what he is going through or if he is not understanding” the content. They explained that, “over the phone, you are really reliant on nonverbal cues that are also nonvisual. So it’s double the challenge.” These mixed results may reflect the level of experience of the counsellors: the counsellor who viewed it to be a positive was more experienced, whereas the counsellor who viewed it as a negative had never provided any counselling before. In addition to the above, the counsellors indicated that sometimes the children’s attention drifted, and that being together in person might have reduced this, or that they would have been better able to identify it.

These mixed results were echoed in the interviews with families. Several families said that they would prefer face-to-face therapy because they thought that children would concentrate better, understand more, and find it easier to express themselves. Both caregivers and children emphasized the benefit of being able to see the other person’s reaction, body language and facial expressions. Children would be less likely to get bored, which was more of a problem for phone therapy when children were aged 12 years or younger. However, most caregivers said that they were comfortable using the phone for sessions and one thought that it was more comfortable for children who are shy than seeing a counsellor face-to-face: “She didn’t see her [the counsellor]... so she was able to speak more than if she saw her face-to-face.”

Despite the above challenges, most of the families were very accepting of and positive about telephone delivery. They often indicated to the counsellors that they preferred it to in-person therapy because of the challenges of attending clinics. A counsellor explained that “there are many challenging things, but it’s [because of] their living conditions more than their acceptance for the phone or not.”

### t-CETA is adapted to potential cultural blocks

t-CETA was specifically adapted for use with Syrian refugee children, as well as for lay person delivery, and the counsellors indicated that they felt that it was well-adapted culturally. They felt well trained and prepared to counsel the children. They found the training particularly useful because of the applied components, including extensive role play: “we apply all the components. We apply it. We do role plays for everything. We cover all the manual. We’ve covered many challenges that may appear.” By having lay counsellors with prior experience of case management/social work with Syrian refugees, t-CETA was delivered by people who knew the context and challenges well. Having telephone delivery and lay counsellors also enabled the counsellors to be more informal with the children than is typical in therapy: “I feel like I’m at ease when I’m working with them because the therapy context in this situation is more human than the clinical private setting … with refugees … it’s friendlier, it’s more accessible for patients” (counsellor).

By adapting t-CETA to the context and having it delivered by local counsellors, the counsellors discussed mental health problems in a locally appropriate way,

I needed to provide therapy in itself but adapt it to the context because … I needed to take into consideration their beliefs, because for example, sometimes if I talked about depression that is relatively severe or they had … suicidal ideation, the parents think of demonic possession or jinn possession and they might … get help from a religious person, whereas me, I have to tread more carefully about this subject.

The counsellors were also careful because of potential stigma around having a mental health problem or seeking counselling. Phone therapy was useful in obviating this stigma and therefore in having higher levels of adherence to therapy, “because it’s basically done in the total privacy of the house. So no one would know that this person is going to a therapist or seeking mental health care” (counsellor). Reducing the “risk of being stigmatized and judged” was viewed as particularly important in reaching families with female children because accessing mental health services could impact girls’ later marital opportunities. To ensure that they did not inadvertently let others know that a child was having therapy, the counsellors had a protocol for how to talk to whoever picked up the phone, and for how to contact neighbours or relatives; “We try to get to know who is in the household, their full names to know if someone responded other than the child, that they know everything or not to see how to respond.” If the counsellor did not know the person who picked up the phone, they explained that they were calling from Médecins du Monde but did not make any reference to mental health services. Overall, having local lay counsellors receive in-depth training and extensive supervision was described by the counsellors as being both effective and appropriate, and as good strategy for identifying and addressing potential cultural issues.

### The relationship between the counsellor and the child and caregiver was extremely important

#### Building rapport and trust

The adaptation to cultural blocks identified by counsellors was reflected in children’s and caregivers’ views about the relationship and rapport between them and the counsellors. This influenced children’s willingness to engage with counselling. Both children and caregivers said that they trusted the counsellor and were made to feel comfortable and relaxed in the sessions. One mother said, “Every week I used to wait for the interview. I’d like talking to her. I feel relaxed and he [child] relaxes.” They highlighted the character and skills of the counsellors, saying that they were knowledgeable, respectful, accommodating, and treated them with attentiveness and care. This had a positive impact on the sessions. The mother of a 14-year-old said that the counsellor:

Gives and takes with the person, and she gives them room to talk and say whatever they want, you know? I felt like when I spoke to her, I relaxed… like the most important thing is she knows how to reach out to a child and knows how to approach each person depending on their age.

It was clear that the counsellors could build rapport and this provided a strong foundation for the counselling, enabling children to talk honestly. Several caregivers said that their child was able to be open with the counsellor, sometimes sharing things that they had not shared with family members including details about traumatic experiences. Children were more likely to accept advice from counsellors: the counsellor “would wake him up, like give him advice… he would hear it from her and not hear it from me” (mother).

Caregivers highlighted that children felt able to talk honestly because the sessions were conducted in private with the counsellor, without the caregiver being present. The counsellor independently spoke to caregivers about the general content of sessions (without giving details), meaning that caregivers were aware of the types of problems their child had raised and the skills they were being taught to address them. This built trust, making it easier for children to speak honestly and for caregivers to trust in the process. A mother explained,

The conversation that would happen between her [counsellor] and Ayman [child], for example she would tell me it but not directly. Like, just so that I have an idea of what my son is bothered from… and everything is private between her and him. And I try not to tell my son so that he keeps opening his heart to her.

Caregivers mentioned that they felt comfortable talking openly to the counsellor and trusted the counsellor’s expertise and advice. For some, the feeling that someone was listening to them and helping was important, while children reported feeling more hopeful and positive about life and supported in dealing with specific difficulties.

#### Challenges to engagement

In some cases, children found aspects of the sessions difficult and it was challenging to engage with the counsellor at the outset. Some initially refused or felt unsure about talking to the counsellor on the phone and needed reassurance about speaking to the counsellor alone. Caregivers mentioned shyness or holding back because of depression as reasons for this. In other cases, the child would get bored or distracted. Children said that some aspects of the sessions were hard, specifically talking about memories of traumatic events. For others, difficulties related to understanding the counsellor and/or the content. An 11-year-old girl said, “I didn’t used to understand anything from her [the counsellor].” This girl did not like using a phone in general. However, some children who initially found it difficult to engage because of shyness got more comfortable with time. For example, two mothers said that their child found the first two sessions difficult but after that they started to enjoy talking to the counsellor.

Some mothers and children said that there were times when they did not understand session content at first, but the counsellor would explain and repeat content until they understood. One mother commented, “Now if I found something difficult, we tell her about it, she makes me understand it directly… like we never shut the session having not understood what we are doing and talking about.” Both children and caregivers conveyed an impression that the counsellors were good at picking up when something was not understood and worked to ensure that content was understood before they moved on.

### The family’s attitude to mental health influences their understanding and engagement with counselling

Children and caregivers’ attitude to mental health varied and this influenced the extent to which they engaged with counselling.

#### Positive attitudes to seeking help

Around half of families reported little stigma or shame: they were keen to seek expert advice, were supportive of therapy, and willing to talk to others outside their family about mental health issues and services. This seemed to contribute to caregivers’ involvement in sessions, children’s willingness to do sessions, as well as families’ overall commitment to therapy. Some drew parallels between mental and physical health and did not see mental health problems as something to be ashamed of, pointing out that “a therapist is exactly like any other doctor” (caregiver). An 8-year-old boy, asked about his reason for doing counselling, drew the link between traumatic memories of events in Syria, his feelings of fear, and his body. One mother emphasized the importance of children talking about psychological difficulties to someone other than their parents, which she said could make them feel safe and listened to, while another said, “it’s not shameful or sinful, something one is learning from.” Some families said that they had shared contact details for the service with others, and several children said that they would share techniques they had learned if they had a friend who was struggling.

Openness to talking about mental health problems related to a greater willingness to seek out help and advice from those perceived to be specialists. The mother of a 9-year-old, said:

I like the educated, I like to take other’s opinion bigger than me you know? … I studied till 6/7^th^ grade… Others have done till baccalaureate or university, Arabic literature. So there are people who still know more than me… So I like this really. If something happened with me, I like to talk about it to someone who knows in this.

Many caregivers encouraged their child to engage with the sessions, with some stating that families have a duty to help children get better. One mother, whose daughter initially found the sessions boring but went on to complete them, explained, “I made her understand that the counsellor is helping her and such, she understood more.” Another highlighted that she and the counsellor both wanted what was best for her son and they worked together to help him. Several caregivers expressed gratitude for the efforts of the counsellors.

Caregivers varied in the way they understood their child’s problems, how much they were involved in sessions, and what they saw as their role in their child’s treatment. One mother showed insight into her daughter’s problems, talking at length about the impact of displacement and ongoing adversity on her daughter’s mental health. Another pointed out that “there are things it’s possible the child doesn’t say [to the counsellor], the mom says it for him,” while a different mother said that she told the counsellor about her daughter’s moods so the counsellor would know that she needs to be patient. One also said that she would also ask the counsellor for advice on dealing with her son’s problems. This suggests that some caregivers saw their role as an ally to the counsellor, engaging with the sessions and working cooperatively to help their child. This included prioritizing the sessions, with one mother saying, “The day she [counsellor] wants to call, if I have something, I postpone it.” Another mother described counselling as “a treatment like any other” that “you have to commit” to. The psychoeducation component of t-CETA emphasizes this, suggesting that at least some families took this on board and committed to ensuring that their child completed the course of counselling.

### Stigma and shame

Other caregivers felt less comfortable disclosing and recommending sessions to others and, in some cases, were less involved in the counselling. For example, a grandmother who largely did not participate in her granddaughter’s sessions said that other people “don’t approve of this thing” and that “it’s secret talk, we can’t say it to anyone.” The child also expressed reluctance to mention counselling to others. One mother said that she would be reluctant to recommend counselling to another family in case they interpreted it as an accusation. She also said that she was initially hesitant about accepting mental health services because of the worry that it meant her son was “crazy”, but accepted when the counsellor reassured her that it was just talking and exercises and did not involve medication.

### Prior experience of mental health services

Several caregivers talked about how the sessions were better than expected. In some cases, this related to previous experience with services for refugees. One mother said, “I thought it was a failing plan… But maybe in my perspective it was a failure because many organizations come and take information and then go.” She seemed surprised that this time there was regular follow up and support. The mother of an 8-year-old boy was initially worried that talking over the phone would not be effective:

At first, I used to say it’s possible he might not benefit on the phone… but on the contrary, he benefitted… Like I was not expecting for him to heal and like for this thing to go from him. Because I was telling you I used to take him to a mental health doctor/psychiatrist and he did not benefit from him… I kept taking him almost two months and he did not benefit, not even a little… Here on the phone, first week he was different to me.

Previous experiences of mental health services, including the use of medication, had shaped families’ expectations about counselling, and this was something that was necessary to address through psychoeducation.

### t-CETA works and is needed

The theme derived from interviews with the counsellors, *t-CETA works and is needed*, overlapped substantially with the theme derived from interviews with families, *t-CETA works for many families but there are remaining challenges in other families*, and so they are reported together.

#### t-CETA works for many children

All three counsellors indicated the utility of t-CETA at treating psychopathology. They were all hugely positive about this treatment from both an efficacy and logistical perspective. The efficacy was unexpected for counsellors, who were initially wary: “Really I was amazed and surprised how you can afford a full service on the phone.” They described multiple success stories of children whose debilitating mental health problems were alleviated through t-CETA. These changes were observed by the counsellors – who indicated that “you can hear it and feel it” – and were also told to them by the children’s parents: “it’s a really, really amazing feeling when the parents said ‘thank you, thank you very much. There is a big change.’ And you are just doing it on the phone.” Despite some challenges, all of the counsellors recommended the use of telephone-delivered therapy in the future, indicating “I think it’s a successful program. I really like it. I hope it will continue again, or if any INGO adopts this program for delivering it again, because it really works.” They indicated that particularly in a context of yearly funding cycles for NGOs, having a short, targeted telephone delivery of therapy for specific issues “is exactly what works.” The counsellors felt that it clearly addressed many of the logistical and cultural needs of the children and families, enabled more sessions to be held each day, allowed for better and more rapid follow up when appointments were missed, and was useful for reaching children and families who were unable or unwilling to access in-person therapy.

Many families were similarly positive about t-CETA, with children and caregivers reporting that they enjoyed the session activities, learned from the counsellor, were able to apply what they learned, and their problems had improved. Several mothers were aware of coping techniques that their child had learned and applied to real life situations. One mother described her daughter employing cognitive restructuring:

if she saw two people laughing, it does not mean that they are talking or laughing about you … for example someone passes by and doesn’t say ‘Hi’, that doesn’t mean he or she is upset with her. They might have other things on their mind or problems or something . . . that have nothing to do with us for example.

Children and caregivers described that the children employed other coping skills taught to them, such as counting to 10 when mad, writing down things that were bothering them, doing activities like drawing or coloring, walking or playing with friends, or helping their mother at home. Some caregivers noted that their child had learned about feelings, how to manage relationships with family and friends, and coping with nightmares.

Caregivers also talked about techniques that they had learned to manage their child’s problems, including positive reinforcement, allowing their child to go out (e.g., to play with friends), and dealing with bed-wetting. Several talked about positive effects such as developing better understanding of their child’s problems and no longer using corporal punishment. The mother of a 9-year-old boy said,

Counsellor Farah spoke to me, she told me ‘You have to go along with him, a little from you, a little from me, we have to help each other’ … I feel that he got a bit relaxed . . . I no longer hit him, I no longer keep him in . . . I let him go outside and stuff, he is well with the kids.

Most families reported at least some improvement of problems across different settings including home, school, and with friends. Improvements were noted as improved sleep and fewer nightmares; reduced fear and focus on traumatic memories; feeling calmer; reduced fatigue; improved play and relationships with siblings and peers; decreased anger and fighting; and finding studying easier. Improvements were sometimes noted by others: “My neighbor saw him, how he improved, some of them are telling me ‘Give us the number to talk’”. Some caregivers also compared the sessions favorably to other mental health services. One mother said that her son had previously seen a psychiatrist and had been given medication but did not show any improvement; by contrast he “benefitted a lot” from speaking to the t-CETA counsellor.

### Individual and contextual factors pose challenges to treatment

Problems remained in some children, sometimes due to issues like physical illness or situational factors that are not addressed by t-CETA. Some children said that they were not applying what they learned in the sessions or they could not remember all the content. In one case, the grandmother of an 11-year-old girl explained, “She has iron deficiency. Iron deficiency makes someone forget … She forgets what the counsellor Farah told her.” Some caregivers reported continued problems in their child, including fear and difficult memories, anger, tired psychological state/depression, disciplinary issues, and relationship problems.

Some children said that while they felt better during the sessions, this did not last. Often this was related to ongoing adversity, with families reporting stress due to financial and housing problems, family members with physical and mental health problems, the situation in Syria, separation from family and friends, and lack of access to school. A 17-year-old described how her father was sick, in pain and had a “broken psychological state”, and her mother and sister were depressed. She no longer had access to school and had to start working, their tent had flooded, and her brother had moved to Germany. The girl explained, “All the sessions were good with the counsellor. Good, but every time I would be optimistic, I would get a bump to take me down lower … I am talking to you and being hopeful but after I close [the phone] I get sad over my dad and his situation.” This girl completed only two counselling sessions before having to drop out to work.

Counsellors noted that because of the major structural and environmental challenges faced by refugees, mental health treatment delivered in any form (in-person, over the phone, or another way) was likely to be limited in its efficacy. They explained that the challenges faced by refugees in Lebanon may cause further mental health problems or reduce the efficacy of treatment, while noting that these challenges are also part of why there is such an urgent need for treatment. The counsellors described how the mental health problems of the children were often triggered by their living situation:

I can definitely work with this adolescent in therapy on so many things, but I have to take into consideration the huge limitations that the environment of this same adolescent is putting on our therapy … even if I, for example, adopted the CBT approach for behavior activation for therapy for depression, and I worked on the cognitive reprocessing, for example, I would still come off a bit as patronizing because for this adolescent, the main trigger for depression is something outside of his control.

They were positive about telephone delivery of therapy but cautioned that major structural changes would also need to occur in order to address children’s mental health needs in the most effective and sustainable way.

This was reflected in some families’ expressed need for financial as well as mental health support. One mother explained,

We are missing a lot. The biggest missing thing for us is financial. So, we see someone come to us and take down our names and tells us ‘we want to speak to you, and we want to comfort you’, we hope that this person gives us money.

This family talked about multiple problems that they faced and the impact this had on their mental health. This is a stark reminder that refugee families are likely to have multiple unmet needs that will impact on the likelihood of treatment success, regardless of the mode of delivery.

## Discussion

The aim of this study was to explore the feasibility and acceptability of delivering psychological treatment to children via telephone, by trained and supervised lay counsellors, in the challenging humanitarian context of informal tented settlements in Lebanon. Thematic content analysis of interviews with counsellors who delivered t-CETA and a parallel analysis of interviews with children who received t-CETA and their primary caregivers provided promising evidence suggesting that this approach is feasible and acceptable, while also highlighting challenges that must be addressed if telephone-delivered therapy is to be more widely adopted in humanitarian crisis contexts. This should be considered alongside preliminary evidence from the same sample suggesting that t-CETA is effective in reducing symptoms of psychopathology (with small-medium effect size) and increases the number of children who complete treatment [23].

### Telephone delivery solves logistical and cultural challenges

Both counsellors and families highlighted the problems that telephone-delivered therapy helped to solve. Substantial overlap in the issues raised by counsellors and families strengthened the validity of these findings. For example, difficulty with travelling to clinics was frequently reported to be a significant barrier to accessing standard mental health services, echoing other reports from this context [9, 10]. A telephone-delivered service made access considerably easier as it avoided the cost of taxis, need for childcare, and safety concerns about women and children travelling alone. This is in line with findings from other settings, where high satisfaction with tele-mental health is based on improved access and convenience, and cost savings on travel, childcare, and work commitments [31]. Notably, t-CETA allowed service provision during nationwide protests in Lebanon in 2019 (17 October Revolution) when many services were unable to provide face-to-face appointments. A substantially greater proportion of children in the pilot RCT who received t-CETA accessed treatment (90%) and completed a course of treatment (60%) compared to those who received face-to-face services (60% and 0%, respectively), showing that perceptions of reduced barriers to attendance translated into meaningful differences in retention [23]. This has clear implications for other situations when mobility is restricted, such as the COVID-19 pandemic and natural disasters.

Crucially, receiving psychological therapy via phone potentially overcame some cultural blocks; where people do not use mental health services because of stigma, receiving therapy in the home removed the need to visibly attend a mental health service. This benefit has previously been noted for adults, where tele-mental health may remove barriers such as stigma as well as potentially allowing people to talk more freely [31].

### Challenges to address

Various new challenges were created by telephone-delivery, which needed to be addressed. This included a phone not always being available at the right time, poor phone signal, and difficulty for some families in finding a quiet place to do the sessions. These problems are pertinent in this context, where families may only have one phone, live in rural areas with poor network coverage, and live in overcrowded accommodation where it can be difficult to achieve privacy. Developing protocols to manage these difficulties reduced disruption to therapy and risks to confidentiality. This included avoiding disclosing the reason for the call before establishing the identity of the responder, working with the caregiver to find and maintain an appropriate place for the child to take the call, and jointly agreeing a plan for what to do if it was no longer comfortable for the child to talk or if the call was cut off [27]. The strategies developed helped to improve the situation over the course of therapy in most cases; however, in some cases there were persistent difficulties, such as background noise. It will be important to determine the suitability of telephone-delivery for individual families, including taking into account their scope to find an appropriate place for the calls. This should be decided on a case-by-case basis rather than assuming that tented settlements are not suitable, as some families were able to maintain a suitable space for the duration of sessions.

Some of the difficulties created by telephone-delivery could be mitigated by the increased flexibility afforded by the approach. For example, it was much easier to rearrange sessions that were missed to later in the same day. Telephone-delivery, including offering some calls during evenings and weekends, resulted in considerably more flexibility than would be available with standard services and so less disruption to treatment flow. Caregivers also talked about feeling more supported when there were regular phone calls to follow up with them, in contrast to clinic-based services they had accessed previously. The ability to flexibly and regularly follow up by phone may act to increase engagement relative to face-to-face services, reflected in the high retention rates seen in the pilot RCT [23].

### Adapting communication for telephone-delivery

Both counsellors and families reported that communication with some children was difficult over the phone. Communication was more challenging without visual feedback, making it harder for some children to maintain concentration and for counsellors to judge if children understood or were engaged with session content. For this reason, paying attention to non-visual cues and developing strategies to engage children was a particular focus of training and supervision [27]. For example, noticing and responding to hesitant or contradictory answers, prolonged silences, frequent topic changes, or changes in background noise that might indicate that the child was moving around or interacting with other people. Several strategies were employed to help engagement, such as shortening sessions, gamifying sessions by turning steps into activities, involving the child in decision-making and planning, and incorporating short verbal games to boost concentration and build rapport. Counsellors also highlighted that it was difficult to help children understand concepts without printed materials or being able to use pen and paper. This was made more challenging because many children had missed formal schooling and were unfamiliar with concepts (for example, what a triangle is, which was used to convey the links between thoughts, feelings, and behaviors). While the counsellors developed alternative strategies – such as describing a line linking concepts or getting children to use their fingers as a rating scale – this required incorporating a prior step before it was possible to cover the core session content. If possible, providing workbooks prior to commencing telephone-delivered therapy could support the process, taking into account the impact of missed schooling and other challenges on displaced children’s literacy [32] to ensure that materials are in an accessible format.

Overall, while both counsellors and families highlighted a range of challenges, in reality this was outweighed by the increased accessibility afforded by telephone delivery in this population. Without this option, it is likely that many children would not have accessed treatment; indeed, of those children receiving face-to-face treatment in the control condition of the pilot RCT, 60% accessed any treatment, and none completed a course of treatment [23].

### Benefits of lay counsellors

The use of trained and supervised lay counsellors to deliver psychological therapies has been identified as a way of addressing the lack of mental health professionals in humanitarian settings [14, 17]. However, the counsellors interviewed for this study highlighted further advantages to their involvement. Being local to the Beqaa region and with extensive experience working with Syrian refugees in case management roles, they were very familiar with the context and challenges that refugees experience. They were able to discuss mental health in culturally sensitive terms and build trust with families. The counsellors reported that they could take a less formal approach than a mental health professional might do; this was also facilitated by the fact that children were receiving therapy in their own home rather than in a clinical setting.

The majority of children and caregivers talked about the comfortable and trusting relationship they had with the counsellors, and the ease with which many of them could talk to the counsellors about their problems, supporting the counsellors’ view that their less formal approach brought benefits. This included children talking about traumatic memories during imaginal gradual exposure to desensitize children to feared memories. It is of note that it was possible to build rapport between counsellors and children (and their caregivers) over the telephone, despite the challenges of communication highlighted above, and that this likely made it easier for children to talk about difficult memories and sensitive issues. Key to the involvement of lay counsellors was regular expert clinical supervision based on the apprenticeship model, involving a local psychotherapist with knowledge of the context and an experienced CETA supervisor based at Johns Hopkins University [26]. This created space to reflect on challenges, jointly develop strategies to deal with them, and ensure that learning from each case was communicated to the wider team.

During this study we employed Lebanese rather than Syrian counsellors. This was because of legal restrictions on Syrians working in Lebanon and because during focus group discussions as part of a linked study, Syrian refugees generally expressed a preference to see Lebanese researchers due to uncertainty about the identity or allegiance of other Syrians. This preference may not apply in other contexts or at other points in time, and the acceptability of using Syrian counsellors should be explored further.

### t-CETA works for many children, but not all

There was agreement between counsellors and families that t-CETA works for many children, with a reduction in symptoms over the course of treatment, a finding backed up by quantitative symptom scores and reports by children, parents, and counsellors recorded as part of the pilot RCT [22, 23]. Some children and caregivers also described continuing to apply strategies that they had learned in real life situations, suggesting that at least some of the effects of the intervention persisted beyond the end of treatment. The success of t-CETA was unexpected to counsellors and some caregivers: several said that they were initially skeptical about whether talking over the phone could really help and were surprised by the outcomes. Research from other contexts has highlighted a discrepancy between clinicians and patients, with clinicians more concerned about establishing therapeutic alliance remotely [33], so it is of note that both counsellors and families in the current study were generally positive about the experience.

Reduced likelihood of effectiveness may relate to practical problems such as not being able to maintain an appropriate place for the calls or because children find it difficult to engage over the phone, and further research will be required to determine which factors (e.g., age, presenting problems, living conditions) might help in decision-making about when to use t-CETA. It should be noted that t-CETA involves caregivers throughout and this also contributes to outcomes. Caregivers were asked to be present at the beginning of each call to confirm consent, and at the end when the counsellor would recap the session content to them so that they could support their child in doing homework. Caregivers were also taught techniques such as cognitive restructuring and it is likely that some were able to use this to address their own problems. Furthermore, some also received the *Parenting Skills* component, which taught them techniques to manage their child’s behavior. Caregivers were required to engage with telephone sessions and support their child in understanding the content and practicing techniques, which may be particularly important for younger children [31]. A mixed-methods analysis of the factors influencing treatment success in t-CETA provides further evidence of the importance of parental support during treatment [22].

There was variation in the degree of support that caregivers provided to children during treatment. A significant barrier to engagement was experiencing stigma about mental health problems and caregivers who reported this were less likely to have participated in sessions. CETA includes a component on engagement and psychoeducation, which explores barriers such as stigma and normalizes current symptoms [34], but this may not always be sufficient. Involving family members, such as fathers, seemed to help in some cases but community-wide interventions to reduce stigma might be necessary alongside individual treatment approaches. One mother made clear that she was less concerned about stigma when she realized that t-CETA is a talking therapy; this was contrasted to medication, which carries with it a higher connotation of being “crazy”. Avoiding a medicalized approach to mental health interventions in this population may reduce the effects of stigma as a barrier to using services.

When t-CETA did not work as well, this often related to unmet needs and structural factors that are not addressed by mental health services. Poverty, poor quality housing, physical illness, family separation, lack of access to education and employment, and numerous other stressors play a significant role in the lives of those living in informal settlements [35]. This level of adversity will likely impact the success of any type of psychological therapy, regardless of the mode of delivery, and this was found to be particularly the case for children with a primary presentation of depressive symptoms linked to current living conditions [22]. A case management approach that aims to address a wider range of needs alongside mental health treatment may be of benefit to individual families, but major structural changes to address the challenging living conditions of refugees are necessary for optimal mental health.

### Strengths and limitations

This study is the first to explore the feasibility and acceptability of delivering psychological therapy via telephone to children in a humanitarian setting. Conducting interviews separately with counsellors, children, and their caregivers enabled us to explore their different perspectives, building a more holistic picture and increasing confidence in the validity of the results. To look at what is possible in environments when both resources and mobility are severely constrained, we used only standard telephone calls and no other supporting materials, providing a baseline for what might be feasible in a range of resource-constrained contexts: the addition of workbooks or videocalls, where the situation allows, would likely facilitate therapy.

This study is limited by a relatively small sample size, though we interviewed all the staff involved in delivering t-CETA (two lay counsellors and their local supervisor) and the majority of children and caregivers who received t-CETA; this included families who dropped out, to avoid biasing the results towards those with more favorable views. Twenty-five interviews were sufficient to draw out similarities and differences in experiences to draw preliminary conclusions from. We had to conduct interviews for this study either online or via telephone due to mobility restrictions at the time of the interviews. This made some interviews more difficult and may have reduced the quality of the data, though it should be noted that participants were familiar with talking about their experiences over the phone due to their experience of t-CETA.

## Conclusions

There is promising evidence to suggest that trained lay counsellors delivering psychological treatments such as CETA via telephone is both feasible and acceptable in the challenging humanitarian context of informal tented settlements in Lebanon. It has the potential to increase access to mental health services in hard-to-reach populations who struggle to use existing services. While many services temporarily relied on remote delivery during the COVID-19 pandemic, there is the potential for tele-mental health to bridge the mental health treatment gap in vulnerable communities on an ongoing basis after the pandemic [36]. Humanitarian settings engender specific challenges compared to high income settings, but with sufficient attention to potential challenges, and high-quality supervision of counsellors to support them in finding solutions, it is possible to deliver therapy via telephone to children as young as eight-years-old. Finally, using lay counsellors – carefully selected, trained, and under expert supervision – to deliver psychological treatment is not only feasible but potentially advantageous in terms of being able to recruit counsellors with the greatest familiarity with the population with whom they are to work. Further research in larger samples, and in a range of settings, will help to establish the potential for these approaches to address the significant gap that exists between the need for mental health treatment and available treatment in resource-constrained settings.

### Supplementary Information

**Additional file 1.** Supplementary Materials.

## Data Availability

Due to the sensitive nature of the data and the potential to identify individuals, original data (audio recordings and transcripts) are not publicly available.

## Abbreviations

CETA: Common Elements Treatment Approach
CBT: Cognitive behavioral therapy
PTSD: Post-traumatic stress disorder
RCT: Randomized controlled trial
t-CETA: Telephone-Common Elements Treatment Approach
UNHCR: United Nations High Commissioner for Refugees
