# Supplementary material for: Feasibility and acceptability of phone-delivered psychological therapy for refugee children and adolescents in a humanitarian setting
Source: Confl Health. 2024 Jan 13;18:7. doi: 10.1186/s13031-023-00565-2 (PMC10787498; doi:10.1186/s13031-023-00565-2)
Supplement: Supplementary file 1 — Additional file 1. Supplementary Materials. [file 13031_2023_565_MOESM1_ESM.pdf]

Supplementary Materials

Contents

1. Inclusion criteria and procedure for the RCT 2
2. Interview guide for interviews with counsellors 3
3. Interview guide for interviews with children and caregivers 6

1. Inclusion criteria and procedure for the RCT

Children and adolescents were eligible for the RCT if they (1) were 8-17 years old at recruitment; (2) lived with a parent or other legal guardian who could provide consent; (3) showed clinically significant mental health impairments by meeting diagnostic criteria for depression, any category of anxiety disorder, post-traumatic stress disorder, or conduct or oppositional defiant disorder; (4) did not have problems for which CETA is inappropriate, such as bipolar disorder, psychosis, or severe suicidal ideation, or problems that would preclude delivery over telephone, such as selective mutism; and (5) the child or caregiver had requested mental health services for the child. Caregivers provided informed consent and children provided assent for participation in the RCT before children were randomly assigned to receive t-CETA or standard face-to-face treatment. One family where the child was randomized to face-to-face treatment withdrew from the RCT because they were unable to attend the clinic and a further four had refused randomization for the same reason. In these cases, t-CETA was offered outside of the RCT to ensure that the child could receive treatment. Families in the RCT and those receiving t-CETA outside of the RCT were eligible for the interviews reported here.

At intake for the RCT, all children met DSM-5 criteria for one or more mental disorders following administration of a structured clinical interview and culturally sensitive diagnostic procedures (Kyrillos et al., 2022). Diagnoses included major depressive disorder, dysthymia, post-traumatic stress disorder, panic disorder, agoraphobia, social phobia, separation anxiety, specific phobia, generalized anxiety disorder, obsessive compulsive disorder, conduct disorder, and oppositional defiant disorder. Additionally, three children were suspected to have ADHD, though diagnosis was uncertain because of the difficulty of obtaining their developmental history in the context of war exposure and displacement. The t-CETA treatment flow was planned for each child based on their presenting problems: for seven children, the primary focus was on trauma symptoms, and for four children, the focus was on depression. Common components on psychoeducation, cognitive restructuring, and finishing steps were planned for all children. Sessions delivered to children were repeated separately with caregivers (when they were able to attend) to ensure they understood what their child had been taught and to enable them to support their child in completing homework between sessions. For five children, caregivers also received a parenting skills component to help them manage their child's behavioral problems.

References

Kyrillos V, Bosqui T, Moghames P, Chehade N, Saad S, Abdul Rahman D, Karam E, Karam G, Saab D, Pluess M, McEwen FS. (2022). The culturally and contextually sensitive assessment of mental health using a structured diagnostic interview (MINI Kid) for Syrian refugee children and adolescents in Lebanon: Challenges and solutions. *Transcult Psychiatry*. https://doi.org/10.1177/13634615221105114

2. Interview guide for interviews with counsellors

Introduction: Before we start, I just want to tell you who I am and what we're going to do.

- My name is XXX and I'm from Queen Mary University of London.
- I'd like to talk to you about providing t-CETA counselling over the phone.
- Because it's a new way of doing counselling which is done over the phone, we don't know if counsellors like it or not, or much about how easy it is to do over the phone. So I want to find out what you thought about it.
- I'm really glad that you've agreed to speak to me today. You talking to us will really help us improve it for other children and families who have t-CETA counselling in the future, as well as for the counsellors who provide it.
- I'm not part of the main research team, but I'm helping specifically with these interviews to help learn more about how the counselling works over the phone.
- I won't tell your colleagues at Médecins du Monde what you said, and neither your name nor any identifying information will ever be associated with any of the quotes. The information from interviews with you and your colleagues will be combined so that any piece of information is not attributable to any one person.
- It's OK to tell me things you *didn't* like about providing the counselling and things that you think *didn't work well* as well as things you *did* like and *did think* worked well. I am particularly interested in what you didn't like or didn't think worked well about t-CETA because if we know this then we can change t-CETA to make it better for children receiving counselling and for the counsellors who provide it in the future. [Check understanding of confidentiality and if they have questions about the interview, confidentiality, etc.]

<u>Interview</u>:

1) So that we can include a range in any papers published from this data, I'd like to ask you a few brief demographic questions. Again, this information will not be tied to any of the things you say later in this interview.
    a. How old are you?
    b. How long have you worked at Médecins du Monde?
    c. What experience do you have as a counsellor before this position?
        i. *Ask about education. Ask about previous positions providing counselling and how long they have been a counsellor.*
2) What was it like providing counselling to Syrian refugee children?
    a. What did you like about working with this particular population?
    b. What was challenging about working with Syrian refugees?
        i. *Probe for aspects of vulnerability, specific types of problems, complex problems, complex family situations, multiple needs, etc.*

        ii. *Probe to see if there are any unique aspects about working with Syrians or refugees.*

3) Had you ever provided counselling over the phone before t-CETA?
4) How did it feel to provide counselling over the phone?
    a. What did you like about speaking to the children over the phone?
    b. What did you find difficult about speaking to children over the phone?
        i. *Prompting for quality of connection, privacy, ability to understand the child, not seeing the child face to face, number of sessions, time sessions were delivered, scheduling of sessions etc.*
    c. What did you like about speaking to the caregiver over the phone?
        i. In what way was it different from speaking to the children over the phone?
    d. What did you find difficult about speaking to caregivers over the phone?
        i. In what way was it different from speaking to the children over the phone?
5) Were they any unique challenges delivering t-CETA aside from it being over the phone?
6) How do you think t-CETA worked for the children that you counselled?
    a. What types of problems did it seem to work well for?
    b. What was the most helpful part of t-CETA, and why?
    c. Are there particular types of problems, or individual children, where it didn't seem to work? If so, what types of cases or problems were more difficult to treat? Why do you think it didn't work so well in these cases?
7) Would you recommend the use of phone counselling by other organisations/counsellors? Why / why not?
8) How do you think providing counselling would have been different if children were coming in to see you in person?
    a. What would have been better?
    b. What would have been worse?
9) Would you have preferred to provide counselling face-to-face, rather than over the phone? And if so, why?
10) How did it feel to provide counselling as a lay provider?
    a. Did you feel confident about doing it? Or apprehensive? Why did you feel like this?
11) Did you have any similar experience before you did this job? If so, what?
12) How was the training you received on CETA and t-CETA?
    a. What was good about it?
    b. What was bad about it? What could be improved, and how?

   c. Are there areas where you think you needed more training? If so, what were they and what training do you think would have helped?
13) What support and supervision did you have while you were providing t-CETA?
   a. What was good about the support/supervision you received?
   b. What was bad about it or could have been improved? How could it have been improved?
   c. Is there anything else that would have helped you when you were providing t-CETA?
14) Is there something you would like to see included or excluded from CETA? If so, what is it and why?
15) Is there anything else that you'd like to add?

Debrief:

- Thank you very much for talking to me today. It's really helped me understand what it was like for you to deliver counselling.
- Like I said at the beginning, we won't tell your colleagues at Médecins du Monde any of the things that you said.
- We will use the things you told us to help make t-CETA counselling over the phone better for other children and for other counsellors in the future.
- So that other people can learn about t-CETA, we will write various reports. These will be papers in scientific journals and reports for organisations like NGOs, government departments (e.g., the Ministry of Public Health), etc. If we use any of the words you said during the interview we won't include your name or any identifying information, so no one will know that it was you. [*Check for understanding about confidentiality.*]
- Do you have any questions about this interview, or about what we do with the information you gave us?

3. Interview guide for interviews with children and caregivers

Note to interviewers. Ensure before proceeding that you have completed the informed consent / assent process (consent form with caregiver and assent form with child), that they understand the purpose of the study, and that they have provided consent/assent. Do not proceed with the interview unless you are in a private space where responses will not be overheard. Ensure that informants understand the principle of confidentiality and are given the opportunity to ask any questions before proceeding. Explain the use of taking notes and data storage procedures and ask the participants' permission to record before continuing. Remind participants that they can refuse to answer any question and that they can stop the interview at any time.
Before you start, ensure you have checked names of caregiver and child, child age and gender, and the approximate dates that the child received t-CETA. Do not check extensive clinical data.

Reminder: Study Goal
The goal of this study is to explore the experiences of children and caregivers who received t-CETA counselling. We want to find out about their experiences – both positive and negative – of the counselling, with a specific focus on phone delivery. We also want to hear their suggestions for what might help improve the approach and make it easier.

Reminder: Useful Probes
***** Tell me more about _____ ? You mentioned, what else would you like to say about.
* What do you mean when you say ____?
* Can you give me an example of _____ ?
* Any more examples?
* Silence
* Echo probe
* "Uh-Huh" or non-verbal probes

Interview with child

<u>Introduction</u>: Before we start, I just want to tell you who I am and what we're going to do.

- My name is XXX and I'm from Queen Mary University of London.
- I'd like to talk to you about the counselling that you had recently. It's the t-CETA counselling that you had over the phone with XXX [name of counsellor; check the child understands it's t-CETA and not the phone assessments that you're talking about].

- Because it's a new way of doing counselling which is done over the phone, we don't know if children like it or not, or much about how it works. So I want to find out what you thought about it.
- You talking to us will really help us make it better for other children who have t-CETA counselling in the future. That's why I'm so happy that you've agreed to talk to me today.
- I'm not part of the counselling team, but a different team who want to learn more about how the counselling works
- I won't tell XXX [name of counsellor] or other people who work at Médecins du Monde anything about what you say. It's really important to me that you are honest and can feel free telling me anything you want so that we can make this program better for other kids. It is OK to tell me things you *didn't* like about the counselling as well as things you *did* like, because we will be putting all of the children's responses together and your names will not be included so no one will know who said what. I am particularly interested in what you didn't like about t-CETA because if we know what you didn't like then we can change t-CETA to make it better for other children. [Check child's understanding of confidentiality and if they have questions about the interview, confidentiality, etc.]

<u>Interview</u>:

1) What was it like to participate in t-CETA counselling?
    a. How did it feel to have counselling?
    b. *Probe for both things the client liked and did not like if necessary*
        i. "What was the best thing about counselling? What was the worst thing about counselling?"
        ii. What did you like about t-CETA counselling? What did you not like?
    c. *Probe for both positive/helpful/favourite and negative/not helpful/least favourite aspects if necessary*
2) What was it like talking to a counsellor over the phone?
    a. What did you like about speaking to the counsellor over the phone?
    b. What did you find difficult about speaking to the counsellor over the phone?
        i. *Prompting for quality of connection, privacy, ability to understand the counsellor, not seeing the counsellor face to face, number of sessions, time sessions were delivered, scheduling of sessions etc.*
    c. *Was there anything that made talking over the phone easier? Harder?*
    d. Did you have any challenges in accessing or using a phone regularly?
3) If one of your friends was having troubles, what would you recommend they do? Why / why not?
4) How do you think your counselling would have been different if you had come in to see a counsellor in person?

   a. What would have been better/easier?
   b. What would have been worse/harder?
5) Which would you prefer in the future, having counselling over the phone or having counselling face to face?
   a. *When child responds probe for why they would prefer that way.*
6) How much do you talk on the phone normally? What else do you use the phone for?
7) Can you tell me more about what was going on in your life and what you were like before starting counselling?
   a. What types of problems did you have?
   b. Why do you think you had the counselling?
8) What types of things did you learn about in your counselling?
   a. Did you use any of those things afterwards? How did you use those things? → *Were they able to use the techniques they learned in other situations?*
9) How did you feel when the counselling finished?
   a. What is different about you now than from when you started counselling? Did anything change?
   b. Probe for how they felt about the end of counselling, as well as for changes in symptoms and if this was maintained over time, i.e., do they still feel better now or have things got worse again?
10) Can you tell me about your counsellor, [counsellor name]?
   a. If necessary, remind the child that everything they say is in confidence and you won't tell the counsellor what they said
   b. What was your relationship like with the counsellor?
   c. Was there anything about your counsellor you liked?
   d. Was there anything about your counsellor you disliked?
11) We want to make the counselling better to help other children in the future. What would you change about the counselling to make it better?
   a. Probe for the child's suggestions; encourage them by reminding them that ideas they have might help other children who have the counselling in the future
   b. Let them know that no idea is silly, you want to hear anything they think of
   c. *Probe for things that made it difficult to do the counselling and things that might make it easier*
12) Is there anything else that you'd like to add?

<u>Debrief</u>:

- Thank you very much for talking to me today. It's really helped me understand what the counselling was like for you.

- Like I said at the beginning, we won't tell XXX [counsellor] any of the things that you said.
- We will use the things you told us to help make t-CETA counselling over the phone better for other children in the future.
- So that other people can learn about t-CETA, we will write reports for other people to read. In these reports we will never mention your name, or your family's name, so no one will know that you had counselling. We might include some of the words you said, but we won't include your name, so no one will know that it was you. [*Check for understanding about confidentiality.*]
- Do you have any questions about this interview, or about what we do with the information you gave us?

Interview with caregiver

Introduction: Before we start, I just want to tell you who I am and what we're going to do.

- My name is XXX and I'm from XXX Queen Mary University of London.
- I'd like to talk to you about the counselling that XXX [child's name] had recently. It's the t-CETA counselling that XXX [child's name] and you had over the phone with XXX [name of counsellor; check the caregiver understands it's t-CETA and not the phone assessments that you're talking about].
- Because it's a new way of doing counselling (over the phone) we don't know much about how it works and if families like it or not. So I want to find out what you thought about it.
- I am so happy that you've agreed to speak to me today because it will really help us make it better for other children and their families who have t-CETA counselling in the future.
- I'm not part of the counselling team, but a different team who want to learn more about how the counselling works.
- I won't tell XXX [name of counsellor] or other people who work at Médecins du Monde anything about what you say. So it's OK to tell me things you didn't like about the counselling as well as things you did like. It's good to tell me about anything that you didn't like, because then we can change it to make it better for other families in the future. [Check caregiver's understanding of confidentiality and if they have questions about the interview, confidentiality, etc.]

Interview:

1) What was it like for you and XXX [child's name] to participate in t-CETA counselling?
    a. *Probe for both things the client liked and did not like if necessary*

        i. What was the best thing about counselling? What was the worst thing about counselling?
        ii. What did you like about t-CETA counselling? What did you not like?
    b. *Probe for both positive/helpful/favourite and negative/not helpful/least favourite aspects if necessary*
2) What did you expect of the counselling when you started? Was the counselling like you expected it to be? If not, in what way was it different?
3) What was it like talking to a counsellor over the phone?
    a. What did you like about speaking to the counsellor over the phone?
    b. What did you find difficult about speaking to the counsellor over the phone?
        i. *Prompting for quality of connection, privacy, ability to understand the counsellor, not seeing the counsellor face to face, number of sessions, time sessions were delivered, scheduling of sessions etc.*
4) What did XXX [child's name] think about the counselling?
        i. How did they feel about speaking to the counsellor over the phone?
        ii. What did they like about it? What did they dislike?
        iii. What did XXX [child's name] find easy about talking on the phone? Or was it difficult? If so, what do you think was difficult about it?
    b. Does XXX [child's name] use the phone to speak to other people? Or do they use the phone in other ways? Can you tell me more about how XXX [child's name] usually uses the phone?
    c. Were there any practical challenges with having the counselling done over the phone?
    d. Is there anything else we should know about what it was like to have the counselling done over the phone?
5) If you had a friend whose child had similar problems what would you recommend they do?
6) How do you think the counselling would have been different if you and XXX [child's name] had come in to see a counsellor in person?
    a. What would have been better?
    b. What would have been worse?
7) Which would you prefer more for XXX [child's name], having sessions on the phone or in person with a counsellor? Why?
8) How much do you talk on the phone normally? What else do you use the phone for?
9) Why did XXX [child's name] have the counselling? What type of difficulties did XXX [child's name] have before starting?
10) Did anything change after the counselling? If so what changed and what do you attribute that change to?

   a. *Probe for whether child's problems improved or not and examples of things that changed / did not change*
   b. *Probe for any techniques they learned that they were able to use in other situations; were they able to do this, did it work?*
   c. *Remind them that's OK to tell you if the counselling didn't seem to work, and that it's important to know what doesn't work as well as what works. If it didn't help with the problems, probe them to see if they have any ideas about why.*

11) How did XXX [child's name] being in counselling affect your relationship with XXX [child's name]?
12) How did your child being in counselling affect your family?
   a. *Probe about practical and emotional issues.*
13) How did XXX [child's name] being in counselling affect your relationships with others in your family or community?
14) How did you feel when XXX [child's name]'s counselling finished?
   a. *Probe for how they felt about the end of counselling, as well as for changes in symptoms and if this was maintained over time, i.e., does the child still seem better now or have things got worse again?*
15) How did XXX [child's name] react when their counselling finished?
16) How did you feel about XXX [child's name]'s counsellor?
   a. What was the best thing about their counsellor? What was the worst thing about their counsellor?
   b. *If necessary, remind the caregiver(s) that everything they say is in confidence and you won't tell the counsellor what they said*
17) What do you think we could do to make the counselling better?
   a. *Probe for the caregiver's suggestions; encourage them by reminding them that ideas they have might help other families who have the counselling in the future; let them know that no idea is silly, you want to hear anything they think of*
   b. *Probe for things that made it difficult to do the counselling and things that might make it easier*
18) Is there anything else that you want us to know?

Debrief:

- Thank you very much for talking to me today. It's really helped me understand what the counselling was like for XXX [child's name] and you.
- Like I said at the beginning, we won't tell XXX [counsellor] any of the things that you said.

- We will use the things you told us to help make t-CETA counselling over the phone better for other children and families in the future.
- So that other people can learn about t-CETA, we will write reports for other people to read. In these reports we will never mention your name, XXX [child's name]'s name, or your family's name, so no one will know that XXX [child's name] had counselling. We might include some of the words you or XXX [child's name] said, but we won't include your names, so no one will know that it was you. [*Check for understanding about confidentiality.*]

Do you have any questions about this interview, or about what we do with the information you gave us?
